# Rapidly Growing ALK‐Negative NTRK3‐Positive Inflammatory Myofibroblastic Tumour of the Lung

**DOI:** 10.1002/rcr2.70427

**Published:** 2025-12-08

**Authors:** Yukitaka Sato, Fumitsugu Kojima, Takeshi Ushigusa, Shinsaku Kabemura, Torahiko Jinta, Toru Bando

**Affiliations:** ^1^ Department of Thoracic Surgery St. Luke's International Hospital Tokyo Japan; ^2^ Department of Pathology St. Luke's International Hospital Tokyo Japan; ^3^ Department of Pulmonary Medicine St. Luke's International Hospital Tokyo Japan

**Keywords:** ALK‐negative, inflammatory myofibroblastic tumour, NTRK3, thoracic surgery, video‐assisted thoracic surgery

## Abstract

Inflammatory myofibroblastic tumours (IMT) are rare mesenchymal neoplasms that account for less than 1% of all lung tumours. Although ~50%–60% of IMT cases are anaplastic lymphoma kinase (ALK)‐positive, ALK‐negative IMT cases present diagnostic challenges owing to diverse pathological features. Here, we report a rare case of a rapidly growing ALK‐negative, neurotrophic tyrosine receptor kinase 3 (NTRK3)‐positive pulmonary IMT in a 46‐year‐old man with a history of pulmonary tuberculosis. A 13‐mm nodule detected during routine screening grew to 18 mm within 1 month. Complete surgical resection was performed by video‐assisted thoracic surgery. Histopathological examination revealed spindle cell proliferation with inflammatory cell infiltration. Immunohistochemistry was negative for ALK; however, molecular analysis identified an ETV6‐NTRK3 fusion gene. The patient remains disease‐free at 9 months postoperatively. This case highlights the importance of comprehensive molecular testing in ALK‐negative IMT and demonstrates that complete surgical resection can achieve excellent outcomes even in rapidly growing lesions.

## Graphical abstract

This report describes the importance of comprehensive molecular testing in anaplastic lymphoma kinase‐negative inflammatory myofibroblastic tumour (IMT) and demonstrates that complete surgical resection can achieve excellent outcomes even in rapidly growing IMT.

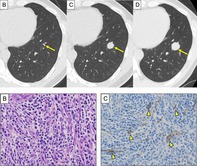

## 1 Introduction

Inflammatory myofibroblastic tumours (IMT) are rare mesenchymal neoplasms that predominantly affect young adults and account for less than 1% of all lung tumours. Approximately 50%–60% of cases demonstrate anaplastic lymphoma kinase (ALK) positivity, whereas ALK‐negative cases present significant diagnostic challenges owing to their diverse morphological patterns [1]. Here, we report a rare case of rapidly growing ALK‐negative, neurotrophic tyrosine receptor kinase 3 (NTRK3)‐positive pulmonary IMT that was successfully treated with surgical resection.

## 2 Case Report

**FIGURE 1:**
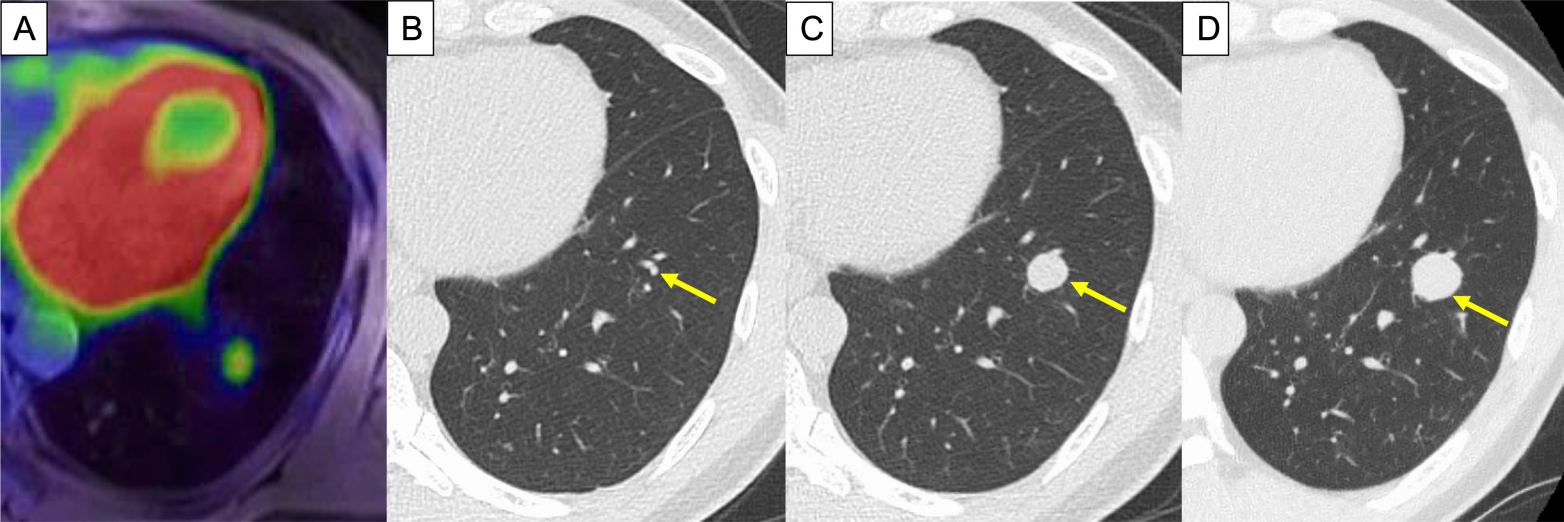
(A) Positron emission tomography‐magnetic resonance imaging (PET‐MRI) shows a solid tumour with fluorodeoxyglucose uptake in the left lower lung. (B) Retrospectively reviewed computed tomography (CT) image reveals a 3‐mm nodule 1 year prior to detection. (C) The patient was referred to our hospital and CT was performed. The CT image reveals a well‐defined 13‐mm nodule in the left S9 segment. (D) A follow‐up chest CT was performed 1 month later. The CT image demonstrates rapid growth to 18 mm, with homogeneous contrast enhancement.

A 46‐year‐old man with a medical history of pulmonary tuberculosis 11 years prior presented with a nodule in the left lower lobe showing increased fluorodeoxyglucose uptake on routine positron emission tomography‐magnetic resonance imaging (PET‐MRI) screening (Figure 1A). He was referred to our hospital, and computed tomography (CT) revealed a well‐defined 13‐mm nodule in the left S9 segment (Figure 1C). Retrospective review revealed a 3‐mm nodule at the same location 1 year earlier (Figure 1B). The patient was asymptomatic, with no fever, weight loss or respiratory symptoms.

A follow‐up chest CT performed 1 month later demonstrated rapid growth of the nodule to 18 mm, with well‐defined borders and homogeneous contrast enhancement (Figure 1D). Previous tuberculosis‐related changes in the right upper lobe remained stable, and a sputum examination for acid‐fast bacilli was negative. Laboratory studies revealed no inflammation or elevation of tumour markers. Bronchoscopic examination of the lesion revealed class II cytology and inflammatory changes on tissue biopsy, without malignant features.

Given the rapid growth pattern raising concern regarding malignancy, surgical resection was performed for diagnostic and therapeutic purposes. A video‐assisted left lower lobe wedge resection was performed. Intraoperative frozen section revealed fibroblastic proliferation with abundant plasma cells and lymphocyte infiltration without malignant features. The sampled mediastinal lymph nodes showed no malignancies.

**FIGURE 2:**
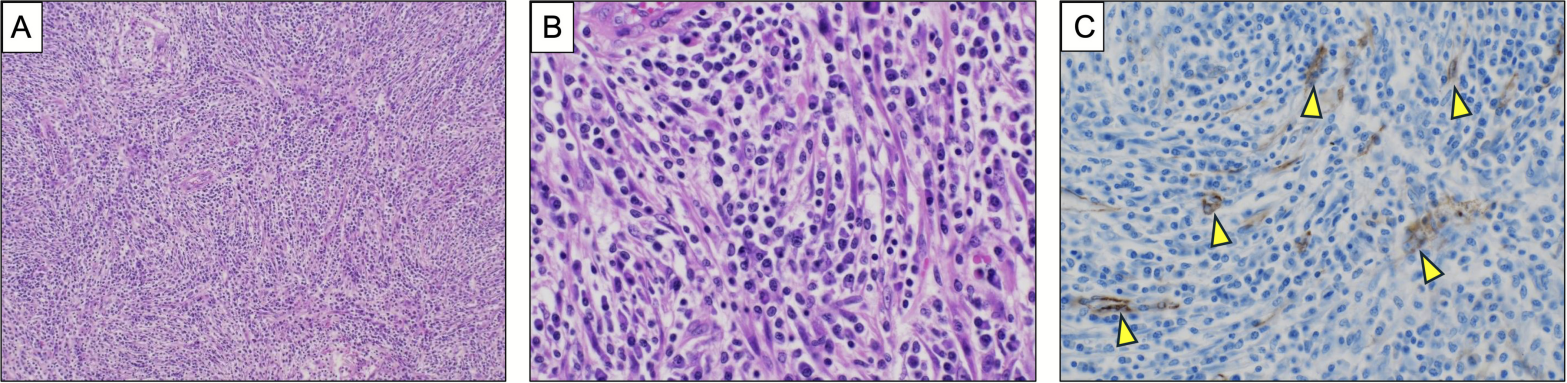
(A) Haematoxylin and eosin staining (HE), low magnification (×10): Spindle cells proliferate in a fascicular pattern with prominent inflammatory cell infiltration. (B) HE staining, high magnification (×40): Spindle cells with eosinophilic cytoplasm and conspicuous nucleoli are observed, accompanied by dense infiltration of lymphocytes and plasma cells in the surrounding stroma. The spindle cells show minimal nuclear pleomorphism and only rare mitotic figures. (C) Immunohistochemistry, high magnification (×40): The spindle cells show cytoplasmic positivity for smooth muscle actin (arrowhead).

The patient recovered uneventfully and was discharged on postoperative day 3. Pathological examination revealed a fascicular proliferation of spindle cells with prominent inflammatory cell infiltration. The spindle cells exhibited minimal nuclear pleomorphism and only rare mitotic figures (Figure 2A,B). Immunohistochemistry showed that the spindle cells demonstrated cytoplasmic positivity for smooth muscle actin (Figure 2C) and negativity for ALK, desmin, CD34 and STAT6. ALK break‐apart fluorescence in situ hybridisation demonstrated no rearrangement. Additional molecular testing was negative for ROS1 and RET but identified an ETV6–NTRK3 fusion, confirming the diagnosis of ALK‐negative IMT. The patient has remained disease‐free for 9 months postoperatively. The postoperative follow‐up is planned for 5 years.

## 3 Discussion

IMT is a rare intermediate‐grade neoplasm of the lungs. Diagnosis can be difficult because imaging and biopsy may not distinguish it from an inflammatory pseudotumor (IPT) or spindle cell sarcoma, particularly in ALK‐negative tumours with broader pathological variability [2]. In the present case, diagnosis was challenging due to the rapid enlargement of an ALK‐negative mesenchymal tumour. However, detection of an ETV6–NTRK3 fusion supported classification as an ALK‐negative IMT rather than an IPT or another spindle cell neoplasm. NTRK3 rearrangements have been identified in ~10%–15% of ALK‐negative IMTs, more commonly in younger patients [3]. Although data are limited to support NTRK alterations themselves as an adverse prognostic factor, histological indicators of aggressive behaviour in IMT—high mitotic activity, hypercellularity and atypical spindle‐cell morphology—have been reported regardless of ALK status [4].

Complete surgical resection with negative margins remains the preferred first‐line management for IMT and reduces the risk of recurrence. For unresectable or recurrent ALK/ROS1 fusion–positive tumours, targeted tyrosine kinase inhibitors, such as alectinib, can be beneficial. More recently, clinical trials have reported that NTRK inhibitors—such as larotrectinib and entrectinib—produce clinically meaningful responses with manageable tolerability in patients with NTRK fusion–positive solid tumours [5]. NTRK inhibitors are therefore considered a viable therapeutic option for patients with unresectable or recurrent NTRK‐positive IMT.

Regarding pathogenesis, immune responses, post‐traumatic reactions and infectious factors, including tuberculosis, have been reported [1]. In the present case, more than 10 years had elapsed since tuberculosis treatment, and there were no imaging or microbiological findings suggestive of reactivation; thus, an association with tuberculosis was considered unlikely.

Although the tumour in our patient showed rapid growth, complete resection led to disease‐free status at 9 months. This case highlights the value of comprehensive molecular testing for ALK‐negative IMT to refine diagnosis and identify potential targeted therapies.

## Author Contributions

Y.S., F.K. and T.J. conceived the idea of this report. Y.S. drafted the original manuscript. F.K., T.U., T.J. and T.B. supervised the writing of the manuscript. T.U. performed the histopathological diagnosis. Y.S., F.K., S.K., T.J. and T.B. performed surgery and perioperative management of the patient. All authors are responsible for the manuscript and approved the final version.

## Funding

The authors have nothing to report.

## Consent

The authors declare that written informed consent was obtained for the publication of this manuscript and accompanying images and attest that the form used to obtain consent from the patient complies with the Journal requirements as outlined in the author guidelines.

## Conflicts of Interest

The authors declare no conflicts of interest.

## Data Availability

The data that support the findings of this study are available on request from the corresponding author. The data are not publicly available due to privacy or ethical restrictions.
